# An Extremely Efficient Silylated Benzensulfonate Flame Retardant for Polycarbonate

**DOI:** 10.3390/ma13163550

**Published:** 2020-08-12

**Authors:** Xiaodong Lu, Qiang Yao, Weihong Cao, Tianbo Tang

**Affiliations:** 1 Ningbo Institute of Materials Technology and Engineering, Chinese Academy of Sciences, Ningbo 315201, China; luxiaodong@nimte.ac.cn (X.L.); caoweihong@nimte.ac.cn (W.C.); tangtianbo@nimte.ac.cn (T.T.); 2 University of Chinese Academy of Sciences, Beijing 100049, China; 3 Key Laboratory of Bio-based Polymeric Materials Technology and Application of Zhejiang Province, Ningbo 315201, China

**Keywords:** polycarbonate, flame retardant, sulfonate salts, silicon

## Abstract

An extremely efficient flame retardant with low water solubility has been developed for bisphenol-A based polycarbonate. Potassium trimethylsilylbenzenesulfonate (KTSS) combining trimethylsilyl and sulfonate groups in its molecule is 7 times less water soluble and 5 times more effective in flame retardancy than potassium benzenesulfonylbenzenesulfonate (KSS), the commercial workhorse for polycarbonate (PC). At a loading of 0.02%, KTSS enables PC to achieve a solid UL-94 V0 rating and a limiting oxygen index (LOI) value of 34.4%, representing an increase of 8.5 units. The extremely high efficiency of KTSS stems from its great migration ability to the burning polymer surface facilitated by trimethylsilyl group, its timely release of active alkaline species that promote the charring process of PC, and the stabilization of char by silicon. In addition to the exceptional flame retardancy, PC/KTSS retains excellent physical properties of PC.

## 1. Introduction

Flame retardant bisphenol-A based polycarbonate (PC) has been widely used in the electronic industry thanks to its balanced physical properties and flame retardancy [1,2,3]. The flame retardancy of PC has been frequently accomplished by incorporating a small amount of sulfonate salts [4,5,6,7]. Particularly, potassium benzenesulfonylbenzenesulfonate (KSS) and perfluorobutane sulfonate (KPFBS) have been commercial workhorses in the fabrication of flame retardant polycarbonate sheet [8,9]. At a loading level of merely 0.1%, both of them enable PC to achieve an impressive UL-94 V0 rating [10,11,12]. 

However, in spite of their high performance, KSS and KPFBS each have their own downsides in the real-world application. KSS suffers from high water solubility, causing concerns that it might leach out in a high-humidity environment. KPFBS, on the other hand, is potentially subject to regulation due to the ban of some structurally similar long-chain per-fluoroalkyl substances [13,14,15]. Thus, an equally or even more efficient flame retardant with low water solubility and a low toxicity would be highly desirable for flame retardant polycarbonate. 

During the search of the next generation of flame retardants for polycarbonate, on the basis of the mode of action of sulfonate salts that takes place in the condensed phase [10], it was surmised that sulfonate salts with great migration ability to the polymer surface upon fire should boast enhanced flame retardancy because a high concentration of sulfonate salts in the surface would significantly facilitate the surface charring process of PC and thus achieve high flame retardancy. To test this concept of the migration enhanced flame retardancy, we have designed a series of molecules possessing silyl and sulfonate groups in their structures. It has been reported that silicon-based flame retardants tend to migrate and accumulate in the burning polymer surface [16,17,18,19]. Thus, by the facile migration of silyl group might sulfonate salts be pulled to the surface leading to enhanced flame retardancy. 

In this study, we report the simplest silyated benzenesulfonate flame retardant for PC with low water solubility and extreme efficiency. By incorporating a trimethylsilyl group in its structure, the new sulfonate salt has not only been demonstrated to be several times more efficient than KSS, but also exhibited a decrease of nearly an order of magnitude in the water solubility. 

## 2. Experiment

### 2.1. Materials

Potassium hydroxide and 1,4-bis(trimethylsilyl)benzene were obtained from Aladdin Biochemical Technology Co. Ltd (Shanghai, China). Carbon tetrachloride and benzenesulfonic acid were acquired from Energy Chemical Chemical Co. Ltd (Shanghai, China). Trimethylsilyl chlorosulfonate was purchased from Aldrich (Shanghai, China). Potassium benzenesulfonylbenzenesulfonate (KSS) was procured from Shouyi Co., Ltd (Yuyao, China). All materials were used without further purification. Polycarbonate (PC, 141R) was purchased from GE plastic (Guangzhou, China). Prior to compounding, the polymer pellets were dried at 110 °C in an oven. Potassium benzenesulfonate (KBS) was obtained by the neutralization of benzenesulfonic acid and potassium hydroxide.

### 2.2. Synthesis of Potassium 4-(Trimethylsilyl)benzenesulfonate (KTSS)

**Scheme 1:**
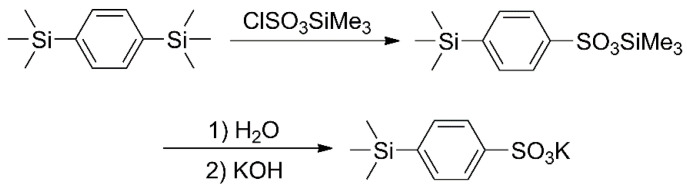
Synthesis of KTSS.

The synthesis of KTSS was carried out according to the reference and is shown in Scheme 1 [20]. Into a 250 mL three-necked flask equipped with a temperature controller, a magnetic stirrer, and a reflux condenser were charged with 8.00 g (0.036 mol) of 1,4-bis(trimethylsilyl)benzene, 6.80 g (0.036 mol) of trimethylsilyl chlorosulfonate, and 50 mL of carbon tetrachloride. After the mixture was stirred at room-temperature under nitrogen atmosphere for 2 h, carbon tetrachloride was distilled out and 10.20 g crude trimethylsilyl 4-(trimethylsilyl)benzenesulfonate was obtained. Then 200 mL water was slowly added to hydrolyze the intermediate to yield 4-(trimethylsilyl)benzenesulfonic acid. This was followed by the addition of 10 mL water containing 2.10 g (0.036 mol) of potassium hydroxide. A white precipitation was obtained. It was recrystallized from water several times. The final yield of KTSS was 83.0%. ^1^H NMR (D_2_O): δ = 7.64, 7.68 (ABq, J_AB_ = 8 Hz, 4H, Ar-H), 0.17 (s, CH_3_-Si, 9H) ppm. ^13^C NMR (D_2_O): δ = −2.34 (Si-CH_3_), 124.44 (C=C-SO_3_K), 133.95 (C=C-Si), 142.68 (C-SiMe_3_), 145.67 (C-SO_3_K) ppm; ^29^Si NMR (D_2_O): δ = −3.52 ppm (Figure S1).

### 2.3. Preparation of Flame Retardant Polycarbonate (PC) Blends

Flame retardant PC blends were prepared via melt compounding at 250 °C in an internal mixer with a roller speed of 50 r/min. The mixing time was 7 min. The materials prepared were transferred into a mold and heated at 250 °C for 5 min then pressed at 10 MPa for 3 min followed by pressing at room temperature for 3 min. The sample plaques obtained were cut into specific test dimensions and stored for further tests. In a similar way, samples of pure PC, PC/KTSS, PC/KSS, and PC/KBS were made. 

### 2.4. Water Solubility

Into 100.0 g of pure water, KTSS or KSS were slowly added under stirring at room temperature until saturation. After standing for 30 min, 50.00 g of the supernatant was taken, filtered, and dried under vacuum to recover the raw material. The water solubility was calculated based on the amount of recovered KTSS or KSS. It was found to be 3.6 g/100 g water for KTSS and 28.7 g/100 g water for KSS at room temperature. The reported value for KSS is 25 g/100 g water [21]. 

### 2.5. Measurements and Characterization

^1^H NMR spectrometry was performed on the Bruker 400 AVANCE spectrometer (Bruker Scientific, Billerica, MA, USA) at 400 MHz using D_2_O as solvent. ^13^C NMR spectrum and ^29^Si NMR spectrum of KTSS were obtained on the same instrument at 100 MHz and 79 MHz, respectively. 

Thermal gravimetric analyses (TGA) of the flame retardants and their PC blends were carried out on 3~8-mg samples using a Mettler Toledo TGA/DSC analyzer (Mettler Toledo Rainin, Columbus, OH, USA) under nitrogen atmosphere at a heating rate of 10 °C/min. TG-IR experiments of the flame retardant blends were conducted on the same equipment. Samples of about 8 mg were heated from 50 to 800 °C under nitrogen (50 mL/min) at a heating rate of 10 °C/min. The spectra were collected every 40 s for 90 min on a Nicolet 6700 infrared spectrometer (Thermo Fisher Scientific, USA). The temperature of the transferring line between TGA and FTIR was set at 200 °C.

The dispersion of KTSS in PC was examined by using a Verios G4 UC energy spectrum scanning electron microscope (SEM-EDX) (Thermo Fisher Scientific, Waltham, MA, USA).

UL-94 vertical burning tests of the flame-retardant blends were conducted on an AG5100B vertical burning tester (Angui Testing Equipment Company, Zhuhai, China) with sample dimensions of 100 × 13 × 3.2 mm^3^ following the protocol of ASTM D3801.

The limiting oxygen index (LOI) tests of the flame retardant blends were evaluated by using a 5801 digital oxygen index analyzer (Yang Yi test Instrument Co., Ltd., Kunshan, China) with sample dimensions of 100 × 6.5 × 3.2 mm^3^ according to the ASTM D2863-97 standard procedure.

Heat distortion temperature (HDT) was measured according to GB/T1634.2-04 in a Zwick/Roell Z020 testing machine (Ceast, Pianezza, Italy).

Tensile properties were tested according to GB/T1040.2-2006 in a 5567 universal material testing machine (Instron, Norwood, MA, USA). The stretching speed was 10 mm/min.

Cone calorimeter test (CONE): The test was performed in a FTT0242 Cone Calorimeter (Fire Testing Technology, West Sussex, UK) according to the standard ISO5660 with a sample size of 100 × 100 × 3.2 mm^3^.

Q-TOF was measured with AB Sciex 4600 Time of Flight Mass Spectrometer (TOF) (AB Sciex, Redwood, CA, USA) on the sample which was obtained by heating KTSS in a DH-900B Carbon Black Content Tester (Innuo precision instruments Co., Ltd, Shanghai, China) at 450 °C for 30 min under nitrogen atmosphere.

The relative concentrations of elements in the top surface of combustion samples of PC and PC/0.1%KTSS were measured by using Axis Ultra DLD X-ray photoelectron spectroscopy (XPS) (Kratos Analytical, Manchester, UK). 

Scanning electron microscopy (SEM) experiments of chars were performed in an EVO18 scanning electron microscope (Carl Zeiss Microscopy, Oberkochen, Germany). Samples for SEM were prepared by low-temperature fracturing and sputtering the surface with gold.

## 3. Results and Discussion

### 3.1. Water Solubility of KTSS 

One concern of KSS as a flame retardant is its high water solubility. Through replacing the hydrophilic sulfonyl group by a hydrophobic trimethylsilyl group, KTSS dramatically reduces its water solubility. As shown in the experimental section, the water solubility of KTSS is 3.6 g/100 g water at room temperature. It is nearly an order of magnitude less soluble than KSS which has a value of 28.7 g/100 g water. Thus, KTSS should be less extractable by moisture in a humid environment. 

### 3.2. Thermal Stability of KTSS

**Figure 1:**
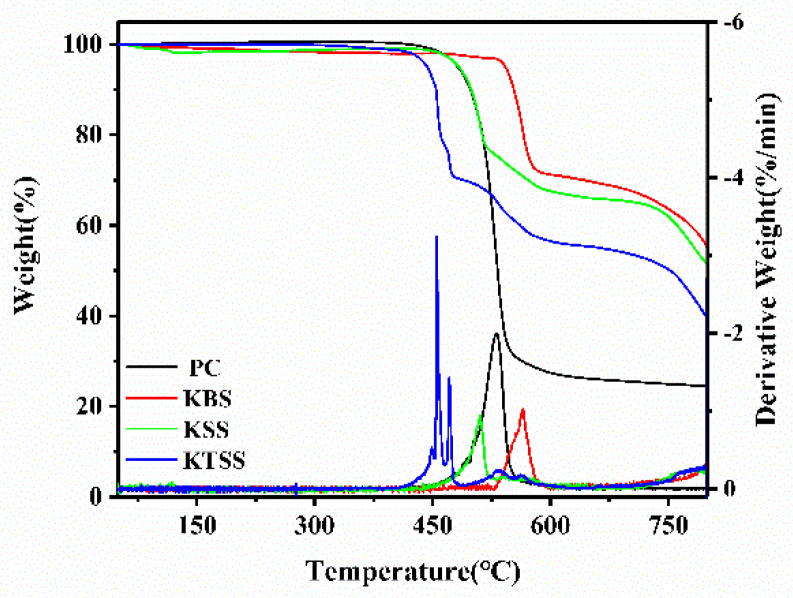
Thermal gravimetric analyses (TGA)/DTA curves of polycarbonate (PC), KBS, KSS, and KTSS under nitrogen.

KTSS possesses a high thermal stability as shown in Figure 1. Although KTSS loses the mass at a temperature lower than KSS, the thermal degradation of KTSS commences at a temperature well beyond the typical processing temperature of PC. The first major decomposition step takes place between 400 and 480 °C with a mass loss of 28.3%. Considering that KBS degrades much later than KTSS, as illustrated in Figure 1, the first step of the thermal degradation of KTSS may involve the trimethylsilyl group. As a matter of fact, a theoretical loss of trimethylsilyl group accounts for 27.3% of the total weight of KTSS, which is fairly close to the actual weight loss in the first stage. Thus, it is presumed that the first stage begins with the bond cleavage of aryl C-Si bond. 

The second degradation step of KTSS happens at 480~600 °C. This is followed by a gradual mass loss until a quick drop beginning around 750 °C. The curve shape of the last two steps is similar to that of KBS, suggesting that they are associated with the sulfonate group. 

### 3.3. Dispersion of KTSS in PC

**Figure 2:**
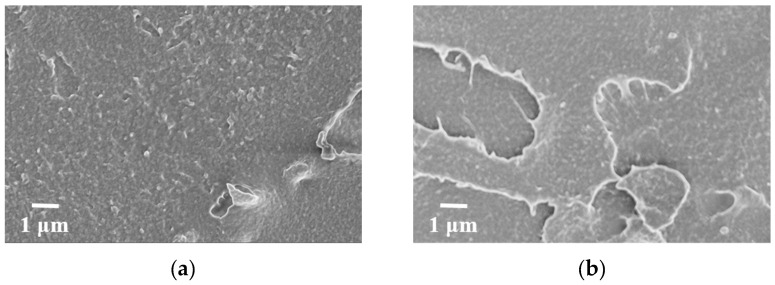
Scanning electron microscopy (SEM) pictures of the top surface of PC (**a**) and PC/1 % KTSS (**b**) 10,000×.

**Figure 3:**
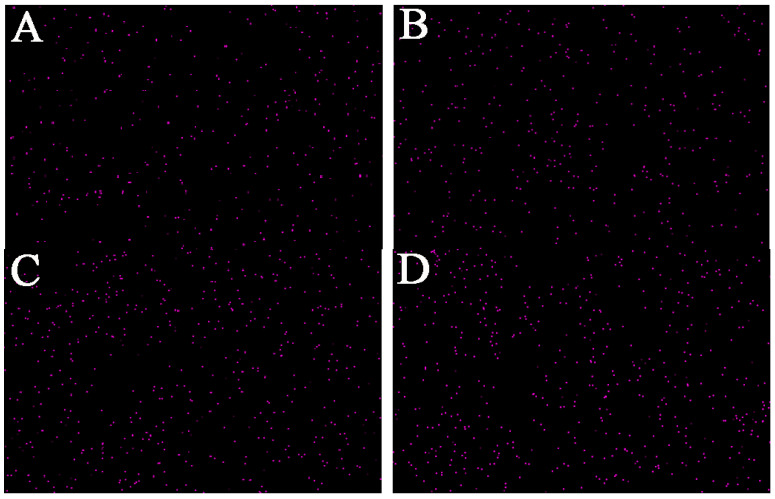
The potassium ion of PC/1 % KTSS at the: (**A**) edge of the outside surface; (**B**) center of the outside surface; (**C**) edge of the cross-section; (**D**) center of the cross-section.

The dispersion of flame retardant in the material could have a huge impact on the flame retardancy and physical properties of the material. Despite its non-melting state, the physical appearance of KTSS in PC cannot be observed even after a magnification of 10,000 times in SEM as shown in Figure 2. PC/KTSS shares the same pattern as PC in terms of surface texture, suggesting that KTSS blends very well with PC. This is further supported by the even distribution of K^+^ obtained from SEM-EDX. The profiles of potassium ion in both the outside surface and the cross section show its random distribution as illustrated in Figure 3. Agglomeration of KTSS is not seen. Thus, KTSS achieves an excellent dispersion in PC. 

### 3.4. Flammability

The results of UL-94 and LOI are shown in Table 1. Remarkably, KTSS shows an extremely high efficiency as a just 0.02% loading enables PC to achieve a UL-94 V0 rating. It is about 4~5 times more efficient than KSS since 0.10% is required for the latter to achieve a comparable performance as evidenced in Table 1. 

In parallel with the excellent UL-94 results, the LOI values of PC are dramatically increased upon the addition of a tiny amount of KTSS. At the 0.02% loading level, KTSS drastically raises the LOI value of PC from 25.9% to 34.4%, a tremendous gain of 8.5 units. This increase nearly doubles the value of 4.4 units achieved with 0.02% KSS. 

What is more, unlike KSS which quickly reaches the peak performance around 0.06%, KTSS continuously improves the LOI values of PC in the tested concentrations. Since sulfonate salts are known to act in the condensed phase [10], the mountain-shaped performance of KSS with its concentrations might suggest that KSS first promotes the char formation of PC at low loadings and then destabilizes the char at high loadings. On the other hand, KTSS possesses silicon which has a stabilizing action on the char [11,22], so it can be made possible for PC to unceasingly attain improved LOI values although the gains decrease at high loadings of KTSS. 

Among the three sulfonate salts, KBS shows a much inferior performance. This should come as no surprise since it is the most stable sulfonate salt as seen in Figure 1. When KBS starts to degrade to release active species, PC has already undergone a significant thermal degradation. Therefore, KBS could not actively interact with the thermal degradation of PC in time and hence has a weak condensed phase action. 

### 3.5. Cone Calorimeter Test (CONE) Analysis of Flame Retardant PC

**Figure 4:**
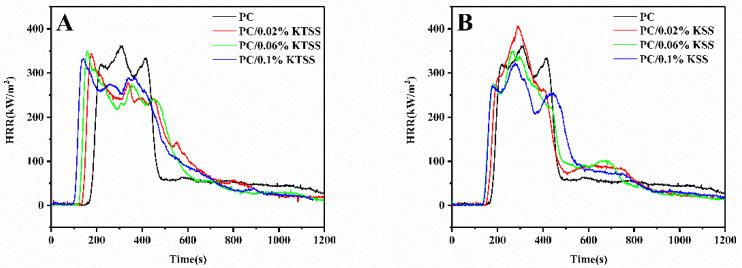
HRR of (**A**) PC/KTSS and (**B**) PC/KSS.

The heat release profiles and the test results of cone calorimetry of flame-retardant PC blends are illustrated in Figure 4 and Table 2, respectively. The virgin PC shows three peaks in the HRR curve with the middle one being the highest. The addition of KTSS even at 0.02% not only considerably reduces the intensity of the last two peaks, but also appreciably shortens the time to ignition. These results suggest a strong chemical interaction between PC and KTSS. This interaction accelerates the production of high-quality char which slows down the degradation of PC as evidenced by the presence of long tails in the HRR curve of PC/KTSS. However, the amount of char is reduced upon the addition of KTSS and PC is almost completely burnt out in the presence of 0.1% KTSS, suggesting that the long term thermo-oxidative stability of intermediate chars is relatively low.

On the other hand, the shape of the HRR curve of PC/KSS at the UL-94 passing level of KSS is hardly changed from that of pure PC. Only the intensities of peaks are lowered, suggesting that high quality char is not produced in the first peak. This is particular apparent at 0.02% of KSS, which even promotes the combustion of PC with an increased PHRR value of the second peak to 407 kW/m^2^, a gain of 12% from that of pure PC. Unlike the TTI of PC/KTSS that shows a unidirectional decrease with loadings of flame retardant, TTI reaches a plateau in PC/0.06%KTSS. This pattern is similar to that of LOI values, reflecting that the stabilization and the destabilization of KSS on the degradation of PC reach a balance. However, similar to KTSS, KSS also reduces the char yield of PC, albeit at a moderate degree. It seems that the faster the formation of char is, the lower the stability of char is. 

**Figure 5:**
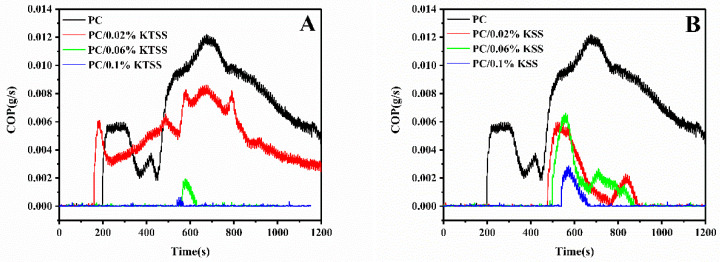
CO production curves of (**A**) PC and PC/KTSS blends, (**B**) PC and PC/KSS blends.

It is noted that both KTSS and KSS significantly reduce the production of carbon monoxide (CO) as shown in Figure 5. For example, the CO release of PC/KTSS or PC/KSS at the 0.02% addition of flame retardant is considerably small compared to that of PC. At high loading levels (0.06~0.1%), KTSS almost completely blocks the generation of CO. The reduction of CO yield can be justified by the action of potassium ion on the change of degradation pathway of phenols. It has been reported by one of us that the thermal degradation of sodium phenolates produces NaOH instead of CO [23,24]. Since PC mainly decomposes to bisphenol A, its interaction with potassium ion would certainly suppress the formation of CO. This result is significant since it means that KTSS can effectively reduce the harm caused by the toxic gas to the human body during the combustion of PC. 

### 3.6. Decomposition of KTSS

**Figure 6:**
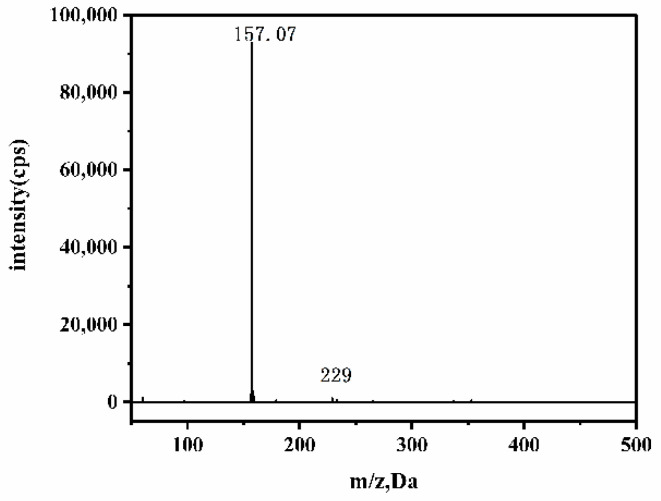
Q-TOF results of KTSS residue obtained at 450 °C under nitrogen.

**Figure 7:**
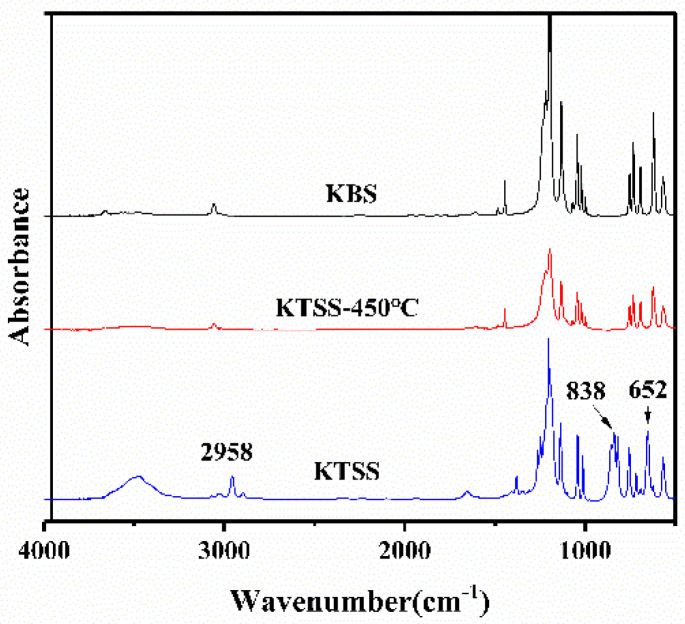
FTIR spectra of KTSS, KBS, and KTSS residue obtained at 450 °C under nitrogen.

**Scheme 2:**
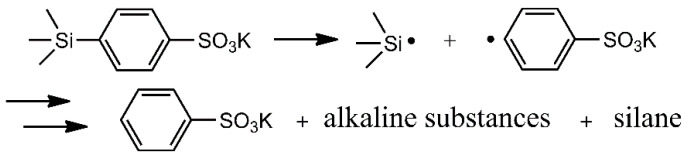
Decomposition of KTSS.

In order to understand the mode of action of KTSS in PC, the decomposition of KTSS was subjected to further examination. Figure 6 shows the Q-TOF result of the sample residue obtained at 450 °C under nitrogen. The small peak at 229.04 in the figure is assigned to KTSS. The huge peak at 157.00 can be attributed to potassium benzenesulfonate (KBS). This is firmly supported by the comparison of IR spectrum of the residue with that of an authentic KBS sample. It can be clearly seen from Figure 7 that all the major peaks of the solid residue are identical to those characteristic to KBS. In addition, the absorptions of 2958 cm^−1^, 652 cm^−1^, and 838 cm^−1^ of KTSS shown in Figure 7 also disappear in the solid residue, indicating the loss of trimethylsilyl group [25]. These outcomes validate the foresaid presumption that the decomposition of KTSS starts with the loss of trimethylsilyl group in the first stage which eventually results in the generation of KBS. The schematic illustration of the decomposition of KTSS is showed in Scheme 2.

This type of degradation of KTSS is highly fascinating since KBS is not as efficient as KTSS or KSS. Thus, it suggests that before the formation of KBS the sulfonated aryl radical must yield active species which can interact with the charring process of PC. It has been hypothesized that the timely formation of alkaline substances from sulfonate salts is critical to achieve good flame retardancy of PC [26]. Although the nature of alkaline substances is not identified in the current study, their presence is consistent with the reduced quantity of CO as evidenced by the measurement of CO release in the cone tests. Thus, it is highly likely that these alkaline intermediate degradation products, instead of the final KBS, promote the charring process of phenols and contribute to the excellent performance of KTSS. 

### 3.7. Thermal Gravimetric Analyses (TGA) Analysis of PC/KTSS

**Figure 8:**
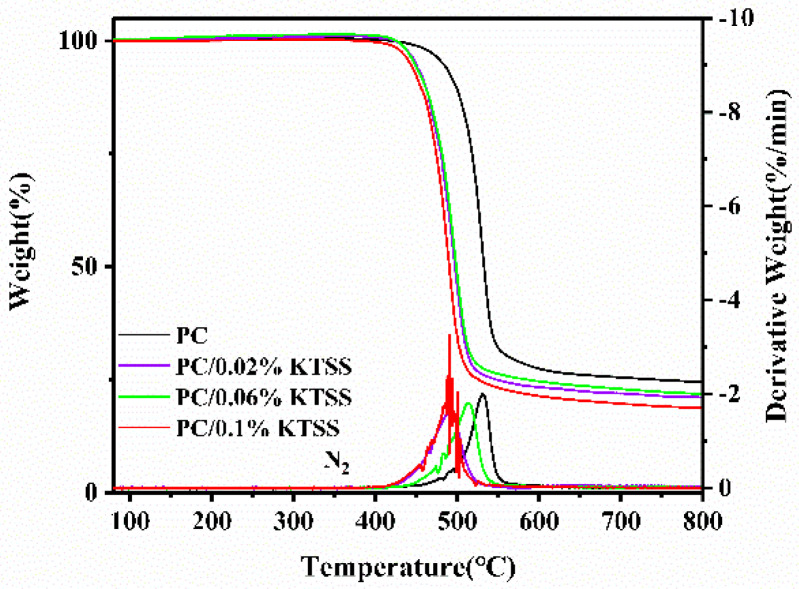
TGA/DTA curves of PC and PC/KTSS blends under nitrogen.

The TGA results of PC and PC/KTSS blends under nitrogen atmosphere are shown in Figure 8 and Table 3. The initial degradation temperature T_5wt%_ of PC is 482 °C, and the T_5wt%_ of PC under different amounts of KTSS are significantly lower than that of PC, indicating that KTSS causes an early degradation of PC. Additionally, the amount of char of PC/KTSS is also lower than that of PC. These results are consistent with those obtained in the cone tests, and suggest that KTSS works as flame retardant by accelerating the formation of char instead of increasing the amount of char. 

### 3.8. TGA-FTIR Analysis of PC/KTSS

**Figure 9:**
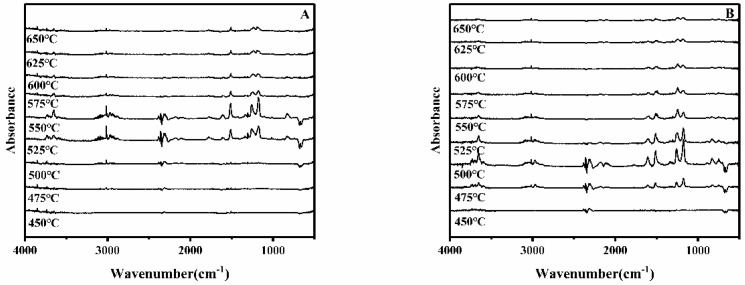
FTIR spectrum of gas phase products: (**A**) PC; (**B**) PC/0.1% KTSS.

**Figure 10:**
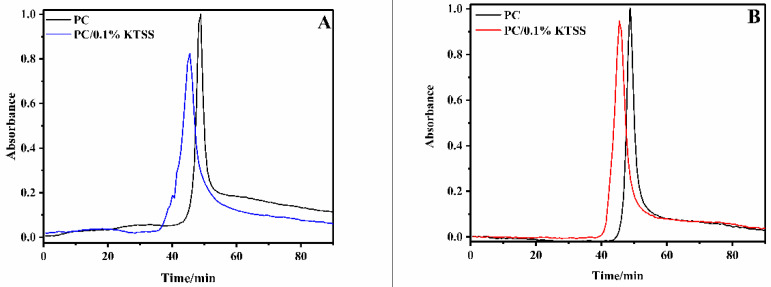
The total absorption intensity of gas phase products: (**A**) gas phase products; (**B**) bisphenol A.

The FTIR spectra of gaseous products from the thermal decomposition of PC and PC/0.1% KTSS blend are shown in Figure 9. In the case of pure PC, the gaseous products are detected at 475 °C and reach a maximum value around 550 °C as illustrated in Figure 9A. For PC/0.1%KTSS, the gaseous products start to appear at 450 °C, 25 °C lower than that of PC. This is line with the destabilization caused by KTSS. The maximum yield of gaseous products also occurs early at 500 °C. In addition, the width of the total absorption intensity of gas phase products becomes wider in PC/0.1% KTSS than PC due to the early degradation of PC as illustrated in Figure 10A. 

Further, a close examination reveals that the gaseous products are essentially same for PC and PC/0.1%KTSS. Bisphenol A and methane are major products in both cases [27,28]. The total absorption of bisphenol A at 1258 cm^−1^ shares a similar pattern in the presence and absence of the flame retardant. The major difference is that bisphenol A from PC/0.1%KTSS emerges at a low temperature as shown in Figure 10B. Thus, KTSS does not change the major types of gaseous products but accelerates the rate of the decomposition of PC. 

### 3.9. X-ray Photoelectron Spectroscopy (XPS) Analysis of Chars

Table 4 shows the XPS results on the residues obtained from the combustion of PC/0.1% KTSS. A high loading of KTSS was used here to increase the accuracy of the measurement. As it can be seen, potassium concentration increases with the ignition time. The final relative concentration of potassium increases by seven-fold, compared with the original level. For sulfur and silicon, their concentrations increase first after the 5 s ignition and then drop after 10 s, suggesting that they accumulate during a short ignition time but decompose upon a long-duration heating. 

Since PC has a much higher thermal stability than KTSS, the initial buildup of Si and S after the 5 s ignition strongly implies the migration of KTSS from the bulk to the surface. Otherwise, the contents of both Si and S should decrease owing to the early degradation of KTSS. The migration of KTSS facilitated by the presence of the trimethylsilyl group is clearly consistent with a great diffusibility of the structurally similar bulky tert-butyl substituted phenyl phosphates [29]. Further, the migration of KTSS is strongly supported by a nearly constant ratio of K/S after 5 s, an expected result from the theoretical consideration. Although the ratio of K/Si increases significantly, this comes without surprise because KTSS begins the degradation with the loss of trimethylsilyl group. 

The accumulation of potassium facilitated by the migration of trimethylsilyl group might be a key to the excellent flame retardancy of KTSS. Since alkali ions promote the char-formation of phenols [23,24], a high concentration of potassium in the surface of PC/KTSS suggests a strong acceleration of char formation of PC. From the results of UL94, KTSS is approximately 5 times more efficient than KSS on the basis of weight. Assuming that the migration ability does not change with the loading levels of KTSS, at 0.02% bulk concentration of KTSS the concentration of K^+^ in the surface should increase 7-fold to 0.020% after the 10 s ignition. Thus, the [K^+^] nearly doubles that of KSS (a value of 0.012%) at 0.1% without assuming the latter’s migration, resulting in comparable values of t_1_ in Table 1. Certainly, this comparison involves many approximations, but it is illustrative enough to demonstrate that KTSS at a low loading level is able to deliver a high concentration of active species in the burning surface comparable with that of KSS at high loading levels so KTSS performs better than KSS. 

### 3.10. Scanning Electron Microscopy (SEM) Analysis of Chars

**Figure 11:**
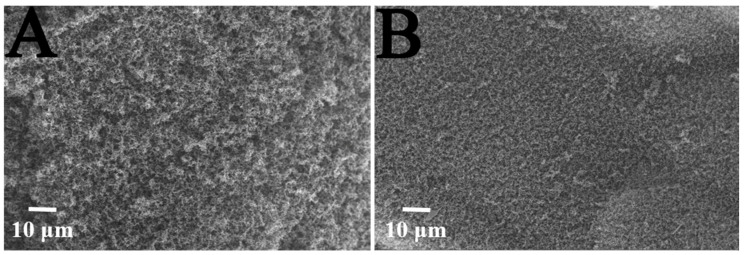

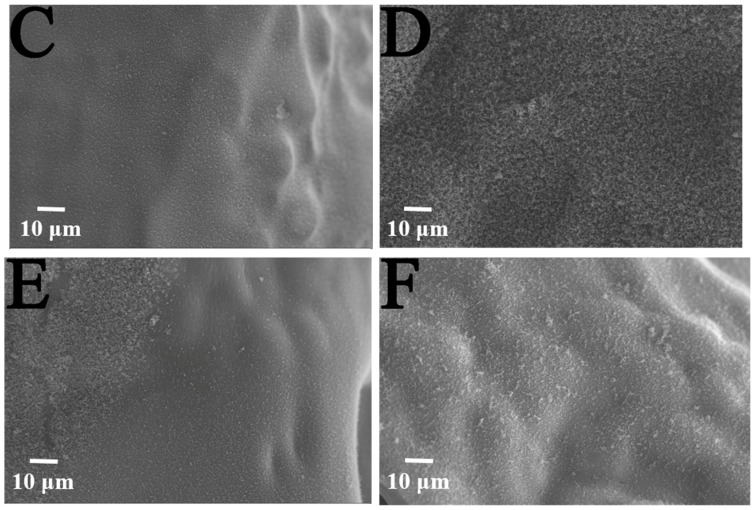
SEM of carbon layer: (**A**) PC, 10 s; (**B**) PC, 20 s; (**C**) PC/0.06% KSS, 10 s; (**D**) PC/0.06% KSS, 20 s; (**E**) PC/0.02% KTSS, 10 s; (**F**) PC/0.02% KTSS, 20 s.

The carbon layer of the surface after the PC combustion is highly porous as evidenced by the appearance of fluffy solids in Figure 11A,B. In the presence of 0.06% KSS, the quality of the surface after the first 10 s ignition is improved and becomes smooth with scattering small fluffy solids, as seen in Figure 11C. However, the carbon layer formed during the first 10 s ignition is not stable and subject to further thermal or thermoxidative degradation in the second 10 s ignition. Thus, the surface becomes highly fluffy again (Figure 11D). This probably leads to the failure of KSS. 

In contrast, both the surface layers of combustion residues of PC/0.02%KTSS are smooth and coherent after the first and the second ignitions (Figure 11E,F). The char is consequently stable toward the further degradation under the test durations of UL-94. The stabilization of char likely comes from the action of silicon, which is known to prevent the char from the oxidation [30,31]. This is supported by the presence of a non-trivial amount of silicon as shown in the XPS analysis of chars. 

### 3.11. Mechanism of Flame Retardant

On the basis of TGA-FTIR, XPS, and SEM results, the mode of action of KTSS is proposed as below: first, KTSS migrates to the surface upon heating as evidenced by the accumulation of S, Si, and K after a short time exposure to flame but before the significant decomposition of PC. It then releases active alkaline species to promote the char formation of PC. The char is stabilized and protected from the further oxidation by silicon which is generated from the degradation of KTSS. Through these coordination processes, KTSS delivers excellent flame retardancy to PC. 

### 3.12. Physical Properties of PC/FR

The physical properties of PC/KTSS are very close to those of pure PC and the effect of KTSS on the physical properties of PC until the 0.1% loading level is minimal as shown in Table 5. Thus, KTSS is essentially inert during the preparation of PC/KTSS blends. 

In the case of PC/KSS, small decreases in the tensile modules are observed with an increased addition of KSS although they may be within errors. However, meaningful differences in HDT upon the addition of KSS can be clearly seen. The HDT values of PC decrease first then increase again with the content of KSS. A difference of approximately 4 °C is noted when KSS is increased from 0.02% to 0.10%. These results probably stem from the accelerated decomposition of PC by K^+^ followed by the crosslinking of PC [32,33]. The larger effect of K^+^ in KSS than KTSS should come from a higher degree of dissociation of K^+^ from the former’s sulfonate group due to the stronger electron withdrawing power of sulfonyl group.

## 4. Conclusions

An extremely efficient flame retardant with low water solubility has been developed for PC. KTSS combining trimethylsilyl and sulfonate groups in its molecule is 7 times less water soluble and 5 times more effective in flame retardancy than KSS, the commercial workhorse for PC. The high efficiency of KTSS comes from its great migration ability to the burning surface facilitated by trimethylsilyl group, and timely release of active alkaline species that promote the char formation of PC, and the stabilization of silicon on the char. Due to a low addition level, PC/KTSS retains excellent physical properties of PC while achieving a solid UL-94 V0 rating at 0.02% KTSS.

In view of the continuous movement toward non-halogenated flame retardants for polymers, our strategy using migration enhanced flame retardancy provides a new tactic to design highly efficient flame retardants. 

## Figures and Tables

**Table 1:** Flame retardancy of PC and its composites with different loadings of FRs.

Sample.	UL94(3.2 mm)			LOI/%
	t_1_/t_2_(s)	D/I ^1^	Rating	
**PC**	1.17/12.90	Y/Y	V-2	25.9
**PC/0.02%KTSS**	0.65/6.33	N/N	V-0	34.4
**PC/0.06%KTSS**	0.97/4.23	N/N	V-0	37.1
**PC/0.1%KTSS**	0.92/3.78	N/N	V-0	38.6
**PC/0.02%KSS**	0.93/8.47	Y/Y	V-2	30.3
**PC/0.06%KSS**	0.95/7.31	Y/Y	V-2	35.8
**PC/0.1%KSS**	0.72/4.42	N/N	V-0	34.4
**PC/0.06%KBS**	0.94/6.91	Y/Y	V-2	
**PC/0.1%KBS**	1.69/6.10	Y/Y	V-2	

^1^ D: dripping; I: ignition of cotton.

**Table 2:** Results of cone calorimetry tests of PC and its flame-retardant blends.

Sample	TTI (s)	PHRR (kW/m^2^)	THR (MJ/m^2^)	t_PHRR_ (s)	Char (wt%)
**PC**	161	361.7	120.4	303	4.2
**PC/0.02%KTSS**	128	344.6	126.9	177	2.6
**PC/0.06%KTSS**	114	349.2	124.8	159	1.1
**PC/0.1%KTSS**	93	332.1	132.5	140	0.8
**PC/0.02%KSS**	145	406.6	125.5	293	2.6
**PC/0.06%KSS**	128	348.8	123.7	263	2.3
**PC/0.1%KSS**	130	319.6	125.0	273	2.6

TTI: Time to ignition; PHRR: Peak heat release rate; THR: Total heat release; t_PHRR_: Time to peak heat release rate.

**Table 3:** TGA data of PC and PC/KTSS under nitrogen.

Sample	T_5wt%_ (°C)	T_10wt%_ (°C)	T_50wt%_ (°C)	T_max_ (°C)	Residues at 800 °C (wt%)
**PC**	482	498	533	531	24.5
**PC/0.02%KTSS**	449	461	497	496	21.1
**PC/0.06%KTSS**	448	460	499	499	21.8
**PC/0.1%KTSS**	442	454	490	491	18.8

T_5wt%_, T_10wt%_, T_50wt%_, T_max_: the temperature at the mass loss of 5%, 10%, 50% and at the maximum rate of mass loss respectively.

**Table 4:** Element contents of PC/0.1% KTSS surface before and after ignition.

Ignition Time	Surface Composition (wt%)					Weight Ratio	
	O	C	S	K	Si	K/S	K/Si
**0 s**	23.00	58.90	1.75	0.32	16.04	0.18	0.020
**5 s**	24.31	40.41	4.94	0.86	29.47	0.17	0.029
**10 s**	23.16	59.28	1.57	2.29	13.73	1.46	0.17

**Table 5:** Tensile properties and heat distortion temperature (HDT) of PC/KTSS and PC/KSS.

Sample	Tensile Modules/10^3^ MPa	Tensile Strength/MPa	Elongation at Break/%	HDT/°C
**PC**	1.95 ± 0.11	62.4 ± 2.1	65.1 ± 3.1	124.1
**PC/0.02%KTSS**	1.93 ± 0.09	66.3 ± 2.0	64.6 ± 5.2	124.5
**PC/0.06%KTSS**	2.04 ± 0.10	71.0 ± 1.0	67.9 ± 6.7	124.7
**PC/0.10%KTSS**	1.98 ± 0.10	68.8 ± 2.1	67.1 ± 5.2	125.9
**PC/0.02%KSS**	1.89 ± 0.08	67.1 ± 2.9	64.1 ± 5.1	121.8
**PC/0.06%KSS**	1.86 ± 0.10	66.4 ± 4.4	70.4 ± 7.2	123.3
**PC/0.10%KSS**	1.81 ± 0.05	66.3 ± 1.3	64.5 ± 4.9	125.6

## Supplementary Materials

The following are available online at https://www.mdpi.com/1996-1944/13/16/3550/s1. **Figure S1:** NMR spectrum of KTSS: (A) ^1^H NMR; (B) ^13^C NMR; (C) ^29^Si NMR.
