# Supplementary material for: An Extremely Efficient Silylated Benzensulfonate Flame Retardant for Polycarbonate
Source: Materials (Basel). 2020 Aug 12;13(16):3550. doi: 10.3390/ma13163550 (PMC7475980; doi:10.3390/ma13163550)
Supplement: Supplementary file 1 [file materials-13-03550-s001.pdf]

materials

MDPI

Supporting information

An Extremely Efficient Silylated Benzensulfonate Flame Retardant for Polycarbonate

Xiaodong Lu [1,2], Qiang Yao [1,2,3,*], Weihong Cao [1] and Tianbo Tang [1]

1 Ningbo Institute of Materials Technology and Engineering, Chinese Academy of Sciences, Ningbo 315201, China, luxiaodong@nimte.ac.cn (X.L.); caoweihong@nimte.ac.cn (W.C.); tangtianbo@nimte.ac.cn (T.T.)
2 University of Chinese Academy of Sciences, Beijing 100049, China
3 Key Laboratory of Bio-based Polymeric Materials Technology and Application of Zhejiang Province, Ningbo 315201, China
* Correspondence: yaoqiang@nimte.ac.cn

Characterization

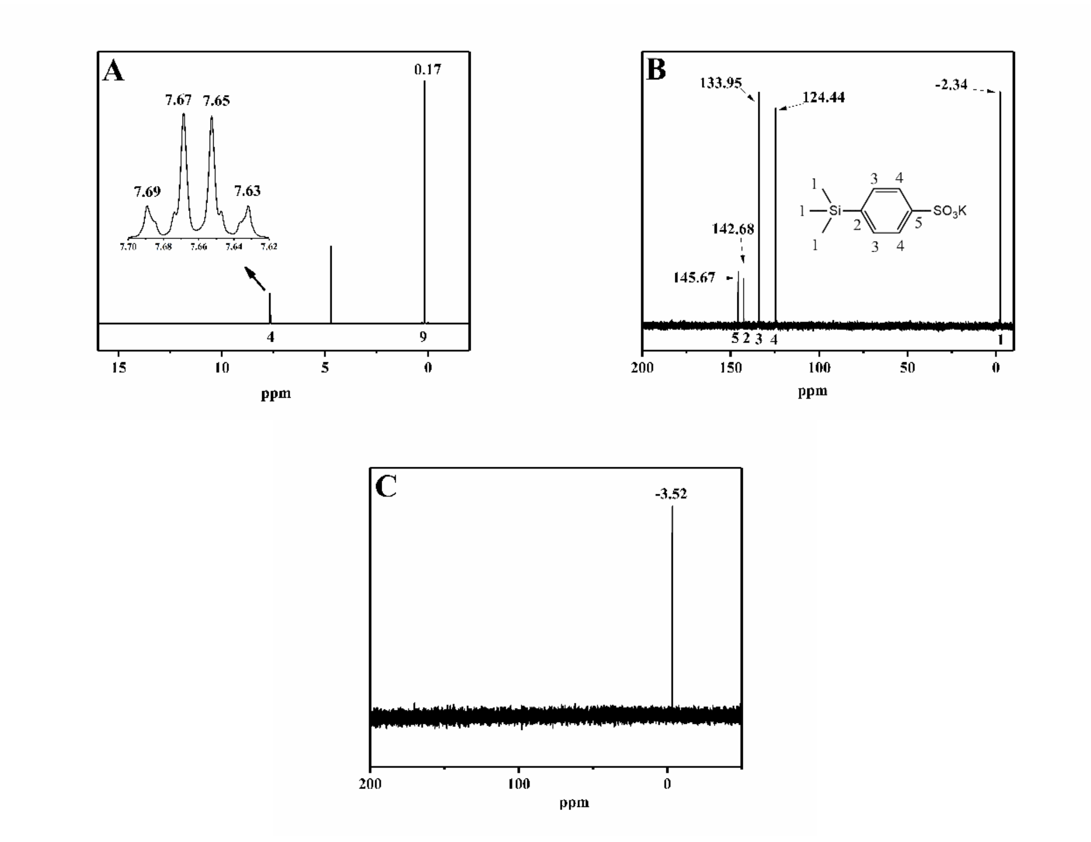

Figure S1. NMR spectrum of KTSS: (**A**) ^{1}H NMR; (**B**) ^{13}C NMR; (**C**) ^{29}Si NMR.

The NMR spectrum of KTSS is shown in Figure S1.

Figure S1A: ^{1}H NMR (D_2O): δ = 7.64, 7.68 (ABq, J_{AB} = 8 Hz, 4H, Ar-H), 0.17 (s, CH_3-Si, 9H) ppm.

Figure S1B: ^{13}C NMR (D_2O): δ = -2.34 (Si-$\underline{C}H_3$), 124.44 ($\underline{C}$=C-SO_3K), 133.95 ($\underline{C}$=C-Si), 142.68 ($\underline{C}$-SiMe$_3$), 145.67 ($\underline{C}$-SO_3K) ppm;

Figure S1C: ^{29}Si NMR (D_2O): δ =-3.52 ppm.
